# Orbital computed tomography biomarkers for the assessment of activity of Thyroid Eye Disease

**DOI:** 10.1371/journal.pone.0349838

**Published:** 2026-08-10

**Authors:** Bo Sook Han, Min Seok Seo, Eun Soo Kim, Ye-Seul Song, Min Joung Lee

**Affiliations:** 1 Department of Ophthalmology, Seoul National University Hospital, Seoul, South Korea; 2 Department of Ophthalmology, Hallym University Sacred Heart Hospital, Anyang, South Korea; 3 Department of Radiology, Hallym University Sacred Heart Hospital, Anyang, South Korea; 4 Big Data Center, Doheon Institute for Digital Innovation in Medicine, Hallym University Medical Center, Anyang, Gyeonggi-do, South Korea; East Carolina University, UNITED STATES OF AMERICA

## Abstract

**Objectives:**

To investigate the relationship between orbital metrics obtained using computed tomography (CT) imaging and inflammatory activity in thyroid eye disease (TED).

**Methods:**

This retrospective study analyzed the medical records and CT images of 85 patients with TED and 15 controls. Orbital segmentation was performed using commercially available software. The volumes and densities of the extraocular muscles (EOMs), intraorbital fat, and lacrimal gland were measured. CT parameters were compared among the control, active TED, and inactive TED groups. Patients were further classified into two subgroups based on disease severity: mild TED and moderate to severe TED, and CT parameters were compared in each severity subgroup.

**Results:**

Patients with active TED showed significantly higher thyroid-stimulating immunoglobulin levels than patients with inactive TED (p < 0.001). Orbital CT analysis revealed that the EOM volume was significantly larger in the active TED than in the inactive TED and control groups (p < 0.001). Additionally, intraorbital fat density was higher in patients with active TED (p < 0.001), whereas intraorbital fat volume and EOM density showed no significant differences between the groups. Lacrimal gland parameters did not differ significantly between the groups. Subgroup analysis based on TED severity also indicated that EOM volumes were larger and intraorbital fat densities were higher in the active TED than in the inactive TED (all p < 0.05).

**Conclusions:**

Orbital CT revealed significant differences in EOM volume and intraorbital fat density between patients with active and inactive TED. These findings suggest that quantitative CT analysis may be useful for assessing TED activity.

## Introduction

Thyroid eye disease (TED) is a complex autoimmune disorder caused by the activation of orbital fibroblasts by autoantibodies against thyroid stimulating hormone receptors. The etiology and pathogenesis of TED are not well understood; however, several histopathological changes are known, including inflammation, adipogenesis, hyaluronan deposition, and fibrosis. The impact of these changes on orbital and periorbital structures leads to the characteristic clinical findings of TED, including proptosis, restrictive myopathy, eyelid and conjunctival swelling and erythema, and dysthyroid orbitopathy.

Several studies have reported that patients with TED typically exhibit a wide spectrum of radiological changes.[1–3] Intraorbital changes such as enlargement of the extraocular muscles (EOMs), expansion of the orbital fat, bony remodeling, engorgement of the superior ophthalmic vein, and enlargement of the lacrimal gland are well known.[4] Expansion of periocular tissues, including brow fat, retro-orbicularis oculi fat, and suborbicularis oculi fat, has also been reported.[2,3] Computed tomography (CT) is the most accessible imaging modality for evaluating orbital diseases.[5,6] In addition to volumetric evaluations, density analyses have been reported. Regensburg et al.[7] investigated the CT densities of the orbital soft tissue and reported that the density of orbital fat was significantly greater in patients with TED than in controls. Recently, CT parameters have been investigated for various purposes, including differential diagnosis of TED, classification of clinical phenotypes, evaluation and prediction of dysthyroid optic neuropathy, and surgical planning.[8–10] However, the relationship between radiological features and TED activity remains uncertain and has not been extensively studied.

Therefore, in this study, we aimed to investigate the relationship between various orbital CT parameters and inflammatory activity in patients with TED and to identify potential imaging biomarker candidates for TED activity.

## Materials and methods

This study adhered to the Declaration of Helsinki, and the study protocol was approved by the Institutional Review Board (IRB) of Hallym University Sacred Heart Hospital. The IRB waived the requirement for informed consent because the study was retrospective and based on a review of electronic medical records. To ensure privacy, all clinical records were anonymized and de-identified prior to the analysis. The raw data are not publicly available due to ethical and legal restrictions regarding patient confidentiality imposed by the IRB. (IRB No 2024-12-001). Data for this research were accessed from 01/02/2025–30/06/2025. The data that support the findings of this study are available upon reasonable request from the IRB of Hallym University Sacred Heart Hospital (contact via cradmin@hallym.or.kr).

### Participants

This study included 85 patients diagnosed with TED who had available CT scans and 15 patients who underwent orbital CT scans for routine checkup or other orbital condition such as contusion, not for TED. A retrospective review was conducted on the medical records of patients who had undergone orbital CT scans at a single oculoplastic center within a tertiary referral hospital in Korea between January 2011 and December 2023. All patients had bilateral TED. In asymmetric cases, the orbit with the more severe involvement was selected to best represent disease burden. When both sides showed similar involvement, the right orbit was chosen for consistency and to avoid inter-eye correlation. The control group consisted of the healthy contralateral orbits of patients who underwent orbital CT for unilateral localized lesions, such as dermoid cysts or cavernous malformation and had no evidence of thyroid-related or other inflammatory orbital diseases. Patients with conditions affecting orbital morphology, such as blowout fractures or prior orbital surgery, were excluded. In the TED group, patients with a history of nonsurgical TED treatments such as immunomodulating drugs or orbital radiotherapy were also excluded.

### TED activity and severity assessment

TED activity was assessed based on 7 point Clinical Activity Score (CAS), with a CAS ≥ 3 considered indicative of active TED. Disease severity was evaluated according to the guidelines of the European Group on Graves’ Orbitopathy [11] and classified into mild and moderate-to-severe groups, with the latter encompassing sight-threatening TED.

### Clinical measurements

The following clinical parameters were reviewed for all enrolled patients: age, sex, TED duration, smoking history, blood thyroid-stimulating immunoglobulin levels, and CAS.

### CT image acquisition

All participants underwent orbital CT with a slice thickness and increment of 2.0 mm Given the long-term retrospective nature of this study, imaging was performed using the institutional scanners active at the time of examination, including a 256-slice MDCT scanner (Brilliance 256; Philips Healthcare), a 64-slice scanner (SOMATOM Sensation 64; Siemens Healthineers), and dual-source scanners (Definition FLASH, Somatom Edge, and SOMATOM Force; Siemens Healthineers). Total body weight-based contrast dosing was used to standardize the contrast media strategy for enhanced CT imaging.[12] Orbital CT scans were strictly performed 50–60 seconds after injection with contiguous coronal slices while the patient’s head was aligned parallel to the Frankfurt plane. The patients were directed to focus on a fixed point. Before the CT scan, all included patients were confirmed to have normal thyroid function test results, including T3, free T4, and TSH, while receiving antithyroid medication. Renal function was also assessed. CT images with severe artifacts and low resolution were excluded.

### Imaging processing and CT measurements

Orbital CT images were analyzed using commercially available semi-automated segmentation software (MEDIP PRO, MEDICALIP Co., Seoul, Korea). Initially, the CT images of each patient were transferred in DICOM(Digital Imaging and Communication in Medicine) format to the CT imaging analysis software. The software provides semi-automatic segmentation of body composition into seven classes. Subsequently, to ensure consistency, the segmentation of the extraocular muscles, intraorbital fat, and lacrimal glands was verified and finely tuned manually by a single experienced oculoplastic specialist (MJL).

**Fig 1:**
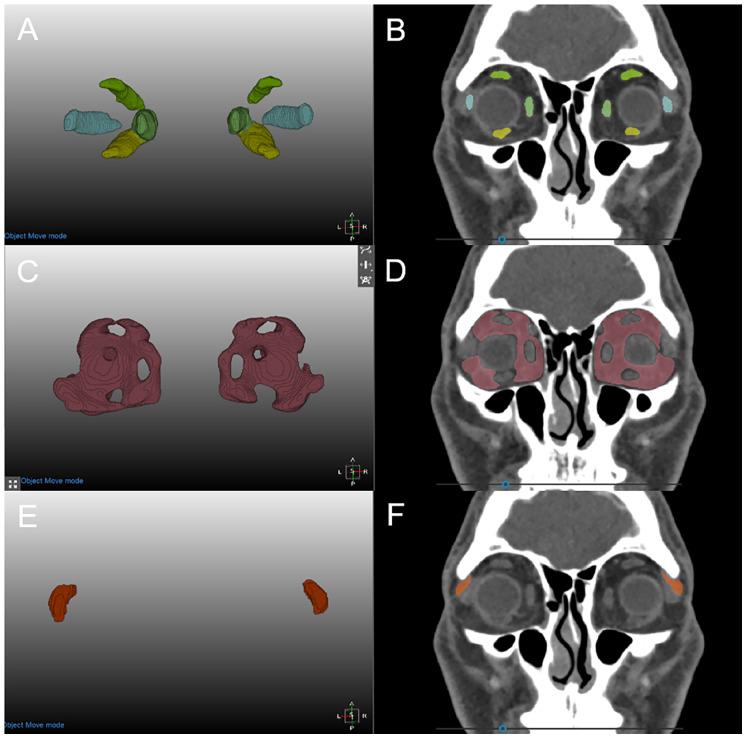
Three-dimensional reconstructed images (A, C, E) with the segmentation of extraocular muscles (B), intraorbital fat (D), and lacrimal glands (F) which were automatically generated with imaging analysis software and finely tuned manually.

After segmentation, three-dimensional reconstructed images were automatically generated, and the volume and density of each structure were automatically calculated. The extraocular muscles include the inferior rectus (IR), medial rectus, superior rectus, and lateral rectus. In addition to the total EOM volume, the individual volumes of the four rectus muscles (superior, medial, lateral, and inferior rectus) were separately calculated and analyzed to assess changes in each muscle in relation to disease activity. The superior rectus muscle and levator palpebrae superioris muscles have an intimate anatomical relationship and are sometimes indistinguishable; these muscles were considered as a single unit. The anterior limit of the bony orbit included the frontal bone, frontozygomatic suture, inferior orbital rim, and anterior lacrimal crest, whereas the posterior limit was defined as the orbital exit of the optic canal. Intraorbital fat was defined as the fat tissue within these boundaries. The volumes and mean densities of orbital structures were measured in cubic millimeters (mm³) and Hounsfield units (HU), respectively (Fig 1).

### Statistical analysis

All statistical analyses were performed using the STATA 18 software (StataCorp LLC, TX, USA). Analysis of variance and additional post hoc pairwise comparisons were performed with Bonferroni-corrected p-values to compare continuous variables among the three groups. Unpaired t-tests were used to test the differences in continuous variables between the two groups. Categorical variables were compared using Fisher’s exact test or the chi-squared test. To identify parameters significantly associated with disease activity, we performed two sequential multivariate analyses: first, a comprehensive multivariable logistic regression model (Model 1) forcing all demographic and clinical covariates (age, gender, GD status, duration of TED, and TSI levels) alongside all radiological parameters. Smoking status was not included because of the substantial proportion of missing data. Second, an optimized stepwise logistic regression analysis (Model 2) to determine the most robust independent predictors of active TED. A receiver operating characteristic (ROC) plot analysis was performed to evaluate the diagnostic performance of the final model. A receiver operating characteristic plot analysis was performed to obtain the area under the curve. Statistical significance was set at p < 0.05.

## Results

A total of 100 patients were included in this study: 85 patients with TED and 15 controls. Among the 85 patients with TED, 45 had active TED, and the remaining 40 had inactive TED (Table 1). The mean age of all patients was 43.84 ± 13.67 years, with no statistically significant differences among the three groups (p = 0.476). The proportion of women was lower in the active TED group than in the inactive or control groups (p = 0.048). Patients with active TED had a shorter disease duration and showed significantly higher CAS scores and TSI values than those with inactive TED (p = 0.037, p < 0.001, and p < 0.001, respectively).

**Table 1:** Clinical characteristics of the study population.

Variable	Active TED (n = 45)	Inactive TED (n = 40)	Control (n = 15)	*p*-value
Age (years, mean±SD)	45.18 ± 14.50	43.88 ± 11.59	40.20 ± 16.03	0.476
Gender, No. of women (%)	22/45 (48.9)	30/40 (75)	9/15 (60)	0.048
TED dur (months, mean±SD)	4.78 ± 4.8	12.15 ± 22.84	–	0.037
Smoking, No. of patients (%)	6/21 (28.6)	16/34 (47.1)	–	0.258
CAS (mean±SD)	3.3 ± 0.7	0.9 ± 0.8	–	<0.001
TSI (SRR%, mean±SD)	402.8 ± 170.8 (n = 44)	224.3 ± 139.5 (n = 36)	–	<0.001

CAS, clinical activity score; dur, duration; SRR, specimen to reference ratio percentages, TED, thyroid eye disease; TSI, thyroid-stimulating immunoglobulin.

### Comparison of Orbital CT parameters among groups classified by activity

Using three-dimensional reconstructed images from orbital CT, we compared the volumes and densities of the EOMs, intraorbital fat, and lacrimal glands among the active TED, inactive TED, and control groups (Table 2). The mean volume of EOMs was significantly larger in patients with active TED than in those with inactive TED or controls (p < 0.001). For intraorbital fat, patients with active TED showed a higher density than patients with inactive TED or the control group (p < 0.001. However, the volume of intraorbital fat and the density of EOMs did not show any statistically significant differences among the three groups. Additionally, the lacrimal glands showed no statistically significant differences in volume or density among the three groups.

**Table 2:** Comparisons of orbital computed tomography parameters among active, inactive TED and control groups.

Variables		Active TED (n=45)	Inactive TED (n=40)	Control (n=15)	*p*-value
Volume (mm^3^, mean±SD)	EOMs	5910.3±2368.1	4233.9±1106.1	3316.0±626.5	<0.001
		A vs I: p<0.001, A vs C: p<0.001, I vs C: p=0.264			
	Intraorbital Fat	18096.9±4469.8	19639.6±4789.9	17088.5±4295.8	0.128
	Lacrimal gland	742.6±266.1	655.1±264.4	715.6±409.1	0.133
Density (HU, mean±SD)	EOMs	71.1±13.0	64.9±14.4	71.5±10.9	0.042
	Intraorbital Fat	-57.3±7.5	-64.6±6.2	-65.3±5.5	<0.001
		A vs I: p<0.001, A vs C: p<0.001, I vs C: p=1.000			
	Lacrimal gland	109.5±20.8	107.1±23.9	117.0±13.2	0.623

A, active thyroid eye disease group; Control group; CT, computed tomography; EOMs, extraocular muscles; HU, Hounsfield unit; I, inactive thyroid eye disease group; TED, thyroid eye disease.

We conducted subsequent multivariate logistic regression analyses incorporating both clinical and radiological parameters to eliminate potential demographic confounding and identify the most robust independent predictors of active TED (Table 3). In the comprehensive multivariable model (Model 1), baseline gender showed no independent statistical association with disease activity (p = 0.565). In the final optimized stepwise model (Model 2), shorter disease duration (p = 0.043), higher serum TSI levels (p = 0.019), larger EOM volume (p = 0.004), higher EOM density (p = 0.034), and higher intraorbital fat density (p = 0.006) were identified as significant independent predictors of active disease. Furthermore, the ROC analysis incorporating these five optimal variables demonstrated an exceptional area under the ROC curve (AUC) of 0.938 (S1 Fig), indicating the outstanding performance of this integrative model in discriminating disease activity.

**Table 3:** Multivariable and stepwise logistic regression analyses for differentiating active and inactive thyroid eye disease groups.

	Model 1: Full Model		Model 2: Stepwise Model	
**Variables**	**Adjusted OR (95% CI)**	***p*-value**	**Adjusted OR (95% CI)**	***p*-value**
**Demographic & Clinical**				
Age	1.054 (0.964 - 1.151)	0.247	–	–
Gender	0.529 (0.060 - 4.637)	0.565	–	–
GD status	0.442 (0.053 - 3.705)	0.451	–	–
Duration of TED	0.871 (0.723–1.049)	0.145	0.868 (0.757–0.995)	0.043
TSI	1.010 (1.001–1.018)	0.023	1.008 (1.001–1.015)	0.043
**Volume (mm**^**3**^)				–
Muscle Volume (MV)	1.002 (1.000–1.003)	0.054	1.001 (1.000–1.002)	0.004
Fat Volume (FV)	1.000 (0.999–1.000)	0.251	–	–
Lacrimal Gland Volume (LV)	1.004 (0.999–1.009)	0.082	–	–
**Density (HU)**				
Muscle Density (MD)	1.053 (0.973–1.139)	0.202	1.063 (1.005–1.124)	0.034
Fat Density (FD)	1.097 (0.940–1.279)	0.240	1.164 (1.044–1.297)	0.006
Lacrimal Gland Density (LD)	1.004 (0.949–1.063)	0.880	–	–

*GD, Graves’ disease; TED, thyroid eye disease; TSI, thyroid-stimulating immunoglobulin. OR, odds ratio; CI, confidence interval.*

*Note: Baseline demographics including age and gender were introduced into the model but were automatically excluded via stepwise selection due to a lack of independent predictive value (p > 0.10).

### Subgroup analysis according to the severity of TED

We classified patients with TED into mild and moderate-to-severe subgroups based on disease severity and analyzed the orbital CT parameters in each subgroup (Table 4). The volume of EOMs and the density of intraorbital fat showed significant differences between active and inactive TED in each severity subgroup (all p < 0.05), similar to the overall patient analysis. However, the density of EOMs did not show significant differences in either the mild (p = 0.192) or the moderate-to-severe subgroup (p = 0.086). Additionally, patients with active TED had a significantly smaller volume of intraorbital fat compared to those with inactive TED in the moderate-to-severe subgroup (p = 0.025).

**Table 4:** Comparisons of orbital CT parameters in severity subgroups.

Variables			Active TED	Inactive TED	*p*-value
Mild subgroup	Volume (mm^3^, mean±SD)	EOMs	4163.2±869.1	3625.3±493.2	0.022
		Intraorbital fat	17646.8±4451.3	17417.9±2963.3	0.849
		Lacrimal gland	761.6±194.7	663.4±236.7	0.160
	Density (HU, mean±SD)	EOMs	73.9±14.0	68.0±13.7	0.192
		Intraorbital fat	-60.0±6.7	-65.3±5.9	0.013
		Lacrimal gland	113.8±22.3	110.0±26.0	0.621
Moderate to Severe subgroup	Volume (mm^3^, mean±SD)	EOMs	7308.1±2259.3	4842.6±1219.9	<0.001
		Intraorbital fat	18456.9±4542.8	21851.4±5284.0	0.025
		Lacrimal gland	727.5±315.1	646.8±295.6	0.385
	Density (HU, mean±SD)	EOMs	68.9±11.9	61.9±14.8	0.086
		Intraorbital fat	-55.2±7.5	-64.1±6.6	<0.001
		Lacrimal gland	106.0±19.3	104.2±21.8	0.767

TED, thyroid eye disease; CT, computed tomography; EOMs, extraocular muscles; HU, Hounsfield unit.

### Individual volumetric analysis of EOMs

Subsequently, we analyzed the volumes of the individual extraocular rectus muscles in the mild and moderate-to-severe TED subgroups (S1 Table). In the moderate-to-severe subgroup, the volume of each of the four extraocular muscles was significantly larger in the active TED group than in the inactive TED group (all p < 0.05). In the mild subgroup, only the volume of the IR muscle was significantly larger in the active TED than in the inactive TED group (p = 0.022).

## Discussion

In our study, we assessed the volume and density of intraorbital structures, including the EOMs, intraorbital fat, and lacrimal glands, using CT images. Our findings revealed significantly larger EOM volumes and higher EOM and intraorbital fat densities in patients with active TED than in those with inactive TED. Stepwise logistic regression and ROC analyses highlighted the predictive value of these measurements for TED activity. Further stratified analyses demonstrated that EOM volume and intraorbital fat density remained significantly different between patients with active and inactive TED, regardless of disease severity.

Imaging studies on various orbital structural changes caused by TED have been actively conducted [13,14], and these findings may be associated with TED disease activity. For example, using magnetic resonance imaging (MRI), Das T. et al. [15] demonstrated that the quantitative T2-relaxation time of EOMs correlates with CAS in TED and is associated with inflammation of the extraocular muscles. MRI offers excellent soft-tissue resolution and various protocols for TED activity assessment; however, its limited accessibility and high cost remain a challenge. Contrastingly, CT is the most commonly used imaging method for diagnosing orbital diseases because of its accessibility, cost effectiveness, and time efficiency.[6] Therefore, this study utilized CT images to explore potential imaging biomarkers of TED activity.

We routinely performed contrast-enhanced CT imaging of patients with orbital pathologies, including TED, to assess the disease characteristics. Currently, studies have reported that the variable incidence of thyroid dysfunction due to iodine contrast medium ranges from 0.05% to 22%.[16–18] In TED patients, there have been occasional reports of adverse effects such as disease reactivation, thyroid storm, or cardiogenic shock following contrast exposure.[19–21] However, in iodine-sufficient countries, contrast-induced thyroid dysfunction is very rare, and when present, it is typically mild, self-limiting, or asymptomatic.[22,23], and Korea is one of the iodine-sufficient countries, where the average iodine intake is about twice the recommended daily intake.[24] Importantly, no significant complications were observed in our study population. To minimize potential risks, we performed contrast-enhanced imaging only in TED patients who were already receiving antithyroid therapy, had stable thyroid function, and normal renal function.

EOM enlargement and orbital fat expansion are the core pathogenic mechanisms of TED, and several studies have demonstrated differences in orbital structural volume on CT between patients with TED and controls.[25] In this study, we conducted volumetric analyses of the EOMs, intraorbital fat, and LG and found that only the EOM volume was significantly larger in the active TED group than in the inactive TED group. To account for the potential confounding effect of disease severity, we performed stratified analyses based on TED severity, as obvious diplopia, suggesting that EOM enlargement, is an important criterion for moderate-to-severe TED. However, even after stratification, a significant difference in EOM volume between patients with active and inactive TED remained in both the mild and moderate-to-severe TED subgroups. Byun et al.[26] investigated the differences in the volume of the orbital structures between patients with active and inactive TED and controls. Similar results were obtained for the volume of EOMs, which was significantly larger in patients with active TED than in those with inactive TED or controls. Similarly, Paniagua et al.[27] reported an association between CAS and the volumes or total EOM and retroorbital fat using MRI.

Only a few studies have investigated the densities of various orbital structures in patients with TED using CT. In this study, the intraorbital fat density was significantly higher in the active TED group than in the active TED group. Regensburg et al. reported that orbital fat density in patients with TED was significantly higher than that in controls.[7] However, they failed to find a relationship between CAS and fat density and speculated that a higher density suggested increased water content in fat or compression of fat cells. Conversely, another study reported no difference in intraorbital fat density in patients with TED compared with the control group, while extraorbital fat showed a significantly higher density in patients with TED than in the control group.[26] These discrepancies may be attributed to the differences in the clinical characteristics of patients with TED, including disease activity. In our study, intraorbital fat density was significantly higher in the active TED group than in both the inactive TED and control groups, whereas no difference was observed between the inactive TED and control groups. These results may be attributed to the use of contrast-enhanced images, which can highlight inflamed tissues. Specifically, the active inflammatory phase of TED is associated with increased vascularity and capillary permeability, potentially resulting in increased contrast enhancement and higher measured attenuation values. Additionally, we observed a trend toward lower EOM density in the TED group than in the control group, although this difference was not statistically significant.

In this study, sequential multivariate regression analyses were performed to identify independent predictors of TED activity while controlling potential confounding factors. Crucially, the full model (Model 1) showed that gender had no independent association with disease activity (p = 0.565), proving that the baseline gender mismatch did not introduce artifactual bias. In the final optimized stepwise model (Model 2), shorter disease duration, higher TSI, larger EOM volume, higher EOM density, and higher intraorbital fat density emerged as significant independent predictors. Although the odds ratios for these continuous metrics appeared close to 1 due to the micro-scale continuous units (mm3 and HU), their cumulative clinical effect across meaningful tissue changes is highly substantial. Most importantly, integrating these optimal serological, clinical, and multi-parameter quantitative CT metrics dramatically improved diagnostic performance, yielding an exceptional AUC of 0.938. This underscores the profound clinical utility of an integrative approach for precise, objective TED activity assessment.

About the LG, neither volume nor density showed a significant difference between patients with TED and controls in our study. However, a few studies have reported that LG volume was significantly larger in the TED group than in the normal population.[26,28,29] These studies hypothesized that antibody-induced inflammation in thyroid eye disease leads to lacrimal gland enlargement, potentially resulting in dry eye. However, results regarding the association between LG size and TED activity have been inconsistent. Rana et al.[30] conducted LG volume measurements using MRI images and reported that LG enlargement was observed in approximately 30% of patients with TED; however, they found no significant difference in CAS between the LG enlargement and non-LG enlargement groups. Contrastingly, the maximal coronal area or degree of LG herniation was suggested as a potential structural change associated with TED activity in a recent meta-analysis paper.[31] We believe that the lack of significant LG changes in our study may be due to the limited soft-tissue resolution of CT. For example, while our study utilized 2-mm slice cuts for CT scanning, the study by Byun et al.[26], which used 1-mm slice cuts for orbit CT, suggests a limitation in the resolution of our CT images.

It is widely accepted that the order of EOM involvement in TED typically follows this sequence: IR, medial rectus, superior rectus, and lateral rectus.[32–36] However, there have been reports emphasizing the significant enlargement of the superior rectus or medial rectus in TED. Bontzos et al.[37] demonstrated that the maximum measured diameter of the medial rectus showed good predictive efficacy for the diagnosis of TED, whereas other researchers reported that the superior rectus/levator was the most affected in TED. However, these studies compared the volume of each EOM in patients with TED and healthy controls. In this study, we compared the volume of each of the four extraocular rectus muscles between active and inactive patients with TED and found that only the IR muscle volume was significantly higher in active patients with TED than in inactive patients with TED, even in the mild TED subgroup. This finding may imply that the inferior rectus muscle is highly sensitive to early inflammatory changes, suggesting that mild TED patients presenting with significant IR enlargement on CT may benefit from closer clinical monitoring for potential activity progression

Our study had several limitations. First, the proportion of women was significantly lower in the active TED group than in the inactive TED and control groups. However, we neutralized this demographic mismatch by performing multivariable-adjusted regression analyses, confirming no significant sex-driven confounding on our findings. Second, while mean HU values provide a representative measure of overall orbital inflammation, they may have a reduced sensitivity for detecting highly localized focal activity. Third, all segmentation corrections were performed by a single observer, and inter- or intra-observer reproducibility was not formally assessed. Although a standardized AI-assisted segmentation workflow was applied to all CT scans, observer-dependent variability cannot be completely excluded. Future studies should evaluate segmentation reproducibility using quantitative metrics such as Dice similarity coefficients and intraclass correlation coefficients. Fourth, because multiple CT scanner models were utilized over the 12-year period, absolute HU measurements could be influenced by specific hardware attributes; nevertheless, this was minimized by strictly adhering to a standardized institutional protocol. Fifth, the control group consisted of unaffected contralateral orbits from patients with localized unilateral orbital lesions rather than healthy individuals due to the retrospective design of this study. Although this approach has been widely adopted in previous orbital imaging studies, contralateral orbit may not be completely equivalent to a truly healthy orbit.[7,26] In addition, subtle effects of unilateral orbital lesions on contralateral CT density measurements cannot be entirely excluded. Lastly, due to the relatively small sample size and the fact that all participants were Korean, these findings should be interpreted with caution when generalizing to other populations. Further studies with larger cohorts are required to validate the potential of these imaging biomarkers.

In conclusion, our study highlights significant findings regarding orbital CT parameters associated with TED activity. The volume of EOMs, along with the density of intraorbital fat and EOMs, showed significant differences between the active and inactive TED groups with good discriminating performance. Furthermore, stratified analysis by severity confirmed that both the volume of EOMs and the density of intraorbital fat were significantly higher in active TED than in inactive TED across all severity levels. These findings highlight the potential of imaging biomarkers for assessing TED activity. The ability to recognize these specific characteristics offers a valuable tool for identifying high-risk patients who warrant prompt clinical evaluation and early therapeutic intervention.

## Supporting information

S1 Fig Receiver operating characteristics curve for differentiating active and inactive thyroid eye disease groups. (TIF)

S1 Table Comparison of the individual extraocular muscle volumes according to TED activity in mild and moderate to severe TED subgroups. (PDF)
