# Supplementary material for: Orbital computed tomography biomarkers for the assessment of activity of Thyroid Eye Disease
Source: PLoS One. 2026 Aug 10;21(8):e0349838. doi: 10.1371/journal.pone.0349838 (PMC13456281; doi:10.1371/journal.pone.0349838)
Supplement: Supplementary file 1 [file pone.0349838.s002.pdf]

Supplementary Table 1. Comparison of the individual extraocular muscle volumes according to TED activity in mild and moderate to severe TED subgroups

Variables (Volume, mm³, mean±SD)		Active TED	Inactive TED	*p*-value
Mild subgroup	Superior Rectus	1181.90 ± 327.31	1004.00 ± 219.94	0.051
	Medial Rectus	1088.39 ± 222.68	989.03 ± 154.18	0.110
	Lateral Rectus	895.96 ± 219.37	845.56 ± 193.27	0.446
	Inferior Rectus	995.25 ± 287.58	823.92 ± 123.93	0.022
Moderate to Severe subgroup	Superior Rectus	1936.05 ± 711.92	1254.35 ± 385.62	<0.001
	Medial Rectus	2173.08 ± 899.80	1264.28 ± 343.93	<0.001
	Lateral Rectus	1224.09 ± 423.61	980.35 ± 287.36	0.027
	Inferior Rectus	1961.93 ± 564.17	1340.80 ± 402.44	<0.001

TED, thyroid eye disease.
